# Limited Myelination Capacity in Human Schwann Cells in Experimental Models in Comparison to Rodent and Porcine Schwann Cells

**DOI:** 10.3390/ijms26136457

**Published:** 2025-07-04

**Authors:** Tak-Ho Chu, Rajiv Midha

**Affiliations:** 1 Department of Clinical Neurosciences, University of Calgary, Calgary, AB T2N 4N1, Canada; thchu@ucalgary.ca; 2 Hotchkiss Brain Institute, University of Calgary, Calgary, AB T2N 4N1, Canada

**Keywords:** porcine, cell culture, dorsal root ganglion, iPSC, motor neuron, Nile red

## Abstract

Schwann cells (SCs) play a crucial role in peripheral nerve repair by supporting axonal regeneration and remyelination. While extensive research has been conducted using rodent SCs, increasing attention is being directed toward human SCs due to species-specific differences in phenotypical and functional properties, and accessibility of human SCs derived from diverse sources. A major challenge in translating SC-based therapies for nerve repair lies in the inability to replicate human SC myelination in vitro, posing a significant obstacle to drug discovery and preclinical research. In this study, we compared the myelination capacity of human, rodent, and porcine SCs in various co-culture conditions, including species-matched and cross-species neuronal environments in a serum-free medium. Our results confirmed that rodent and porcine SCs readily myelinate neurites under standard culture conditions after treatment with ascorbic acid for two weeks, whereas human SCs, at least within the four-week observation period, failed to show myelin staining in all co-cultures. Furthermore, we investigated whether cell culture manipulation impairs human SC myelination by transplanting freshly harvested and predegenerated human nerve segments into NOD-SCID mice for four weeks. Despite supporting host axonal regeneration into the grafts, human SCs exhibited very limited myelination, suggesting an intrinsic species-specific restriction rather than a cell culture-induced defect. These observations suggest fundamental differences between human and rodent SCs and highlight the need for human-specific models and protocols to advance our understanding of SC myelination.

## 1. Introduction

Schwann cells (SCs) are the supporting glial cells in the peripheral nervous system. Under physiological conditions, SCs ensheathe or myelinate axons to provide support and facilitate signal propagation. Under pathological conditions, such as after nerve injury, SCs undergo a mesenchymal cell-like transition into a repair phenotype during Wallerian degeneration [1], secreting a plethora of trophic factors to support neuronal survival and axonal growth. Due to their neuro-regenerative properties, SC-based therapies have been investigated in clinical trials for spinal cord and peripheral nerve injuries [2,3]. While extensive knowledge has been gained from studying rodent SCs, an increasing number of studies are focusing on human SCs due to the accessibility of SCs from diverse sources.

The transition from rodent SCs to human SCs in preclinical studies is crucial and essential, as human SCs exhibit distinct properties compared to their rodent counterparts under experimental conditions. Strategies effective for rodent SCs may not translate directly to human SCs. For example, glial fibrillary acidic protein (GFAP) is a well-established marker for rodent SCs. It is expressed in cultured rodent SCs, non-myelinating SCs in naïve nerves, and dedifferentiated SCs in injured nerves. However, this classic marker is either absent or expressed at a low level in early passages of human SCs harvested from normal nerve biopsies, only reappearing in later passages [4,5]. This observation is consistent with our experience in human nerve- and skin-derived SC cultures [6].

Similarly, Weiss et al. reported that some human SCs exhibit senescence-related features, such as β-galactosidase positivity and cell body enlargement, as early as the second passage [5]. This is in stark contrast to rat SCs which can sustain proliferation with as many as 50 passages [7]. Monje et al. further compared the species-specific differences between rodent and human SCs using high-solution genomic analysis and various cell-based assays [8]. They showed that human SCs require neuregulin for proliferation whereas rodent SCs can thrive with serum alone or with additional growth factors such as platelet-derived growth factor, insulin-like growth factor, and fibroblast growth factor [8]. Additionally, while high levels of cyclic adenosine monophosphate (cAMP) drive myelin gene expression in rodent SCs, cAMP treatment does not induce p75 downregulation or upregulate myelin-associated markers in human SCs [8,9].

These findings highlighted some of the key differences between rodent and human SCs. Another major difference is the ability to demonstrate myelin formation—a critical functional readout for SCs. Numerous studies and established in vitro protocols have shown that rodent SCs robustly myelinate rodent or human neurite with the addition of ascorbic acid, and this myelination is further enhanced with addition of nerve growth factor [10,11]. Human nerve-derived SCs have also been shown to myelinate axons after transplanting into injured immunocompromised rodent models [12,13], or when co-cultured with rat sensory neurons [14], identified with the use of human-specific P0 (monoclonal antibody 592) and HNK-1 antibodies in early studies. A recent study also reported successful myelination of human sensory neurons by human pluripotent stem cell-derived SCs in co-culture and following transplantation into immunosuppressed adult Sprague Dawley rats after sciatic nerve crush [15]. However, most other studies failed to observe human SC myelination. For example, despite their ability to support axonal growth and promote functional recovery after transplantation of nerve-derived SCs into contused spinal cords of immunocompromised rats, no myelination by human SCs can be observed [16]. Other co-culture studies of human nerve-derived or human neural crest cell-derived human SCs with rodent dorsal root ganglia (DRG) also showed incompetency to associate with axons [8] or merely demonstrated SC–axon contact—a pre-requisite for myelination [17]. Similarly, our previous studies using a combination of a human mitochondrial marker and the myelin-associated protein marker periaxin revealed only rare instances of human SCs myelinating rodent axons after transplantation into injured sciatic nerves in SCID mice [6]. Together, convincing evidence of human SCs myelinating neurites of either human or rodent origin in experimental models is still lacking.

Our study aimed to confirm the deficiency in myelination of human SC co-cultures by comparing the culturing conditions with rodent SC co-cultures. In addition, we harvested adult SCs and DRG sensory neurons from pigs, a species closer to humans in terms of physiology and genetic composition than rodents [18]. To further investigate whether cell culture manipulation affected the ability of human SCs to myelinate neurites, we transplanted freshly acquired and predegenerated human nerve fascicles, where human SCs were in their native environment, into NOD-SCID mice. Our study underscored a masked function of human SCs that warrants further investigation.

## 2. Results

### 2.1. Rodent but Not Human SCs Myelinated Neurites in the Presence of Ascorbic Acid

**Figure 1:**
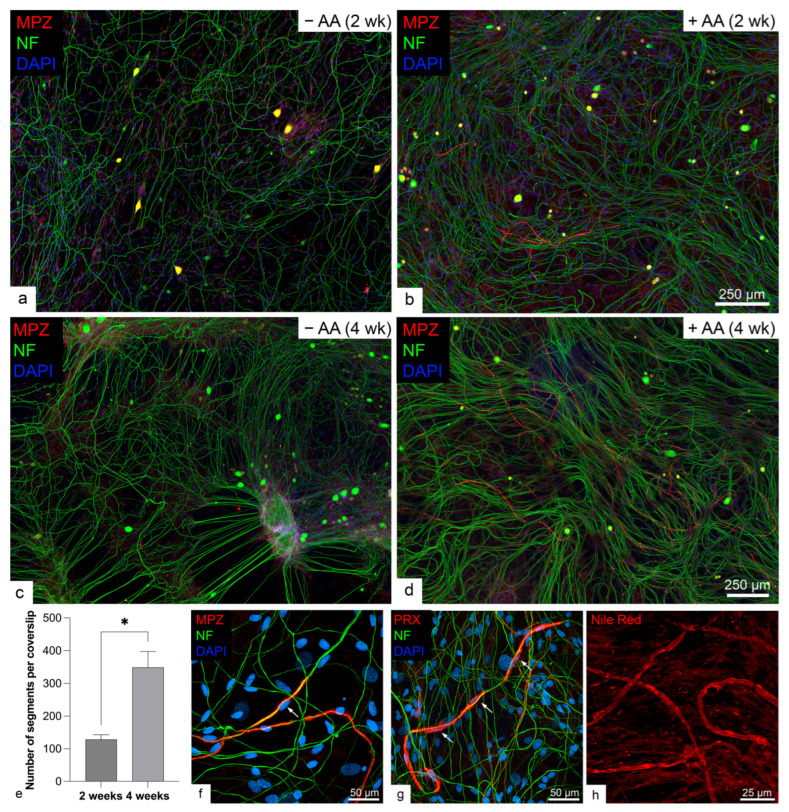
Robust myelination in rat Schwann cell (SC) and rat dorsal root ganglion (DRG) cell co-cultures supplemented with ascorbic acid (AA). A neurofilament (NF) antibody was used to label neurites and DAPI was used to counterstain cell nuclei. No myelin staining is observed in 2- or 4-week co-cultures without ascorbic acid (**a**,**c**), whereas myelination is evident in co-cultures with ascorbic acid (**b**,**d**). Quantification showed a significantly higher number of myelin segments in 4-week compared to 2-week co-cultures grown on 12 mm coverslips in 24-well plates ((**e**), *, *p* < 0.05, *n* = 3, *t*-test). In 2-week rat SC and rat DRG co-cultures treated with ascorbic acid, representative staining from separate samples (**f**–**h**) shows that myelin protein zero (MPZ) staining (**f**) highlighted myelin segments of SCs (arrow), while periaxin (PRX) staining (**g**) labeled both myelin segments and myelinating SC bodies (arrows). Nile red, a lipophilic dye, confirms the lipid-rich nature of the myelin sheath (**h**).

We first confirmed that the serum-free myelination medium formulation supported myelination in rat SCs co-cultured with rat DRGs in the presence of ascorbic acid. After two weeks of co-culture without ascorbic acid, no myelin protein zero (MPZ)-positive staining was found (Figure 1a). In contrast, in the presence of 50 µg/mL ascorbic acid, myelin segments stained positive for MPZ can be observed (Figure 1b). Similar results were found after four weeks of co-culture; no myelin was detected in co-cultures without ascorbic acid (Figure 1c), whereas increased myelin staining was present in co-cultures with ascorbic acid (Figure 1d). Quantification of myelin segments in ascorbic acid-treated co-cultures seeded on 12 mm coverslips in 24-well plates showed significantly higher numbers of myelin in 4-week compared to 2-week co-cultures (Figure 1e, 349 ± 49 vs. 128 ± 15 segments, *p* = 0.012, *t*-test). To confirm myelin formation, we further labeled the co-cultures with another myelin-associated protein marker, periaxin (PRX), and a lipophilic dye, Nile red, which we have previously used to identify myelin in vitro and in vivo [19]. Both myelin antibodies labeled myelin, and we would use them interchangeably in subsequent experiments depending on species. However, compared to MPZ labeling which only showed myelin segments (Figure 1f), PRX also labeled the cytoplasm of the myelinating SCs (Figure 1g). Segments with strong Nile red labeling confirm the presence of lipid-rich myelin in the rodent co-culture (Figure 1h).

**Figure 2:**
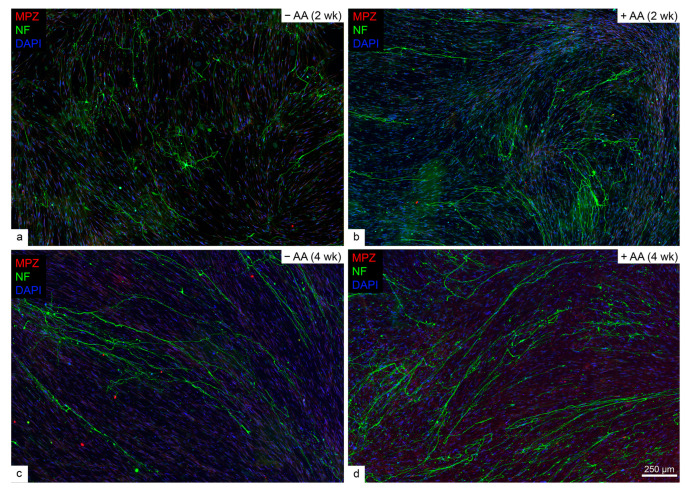
Human Schwann cells do not myelinate human iPSC-derived motor neurons. No myelin staining is observed in either 2- (**a**,**b**) or 4-week co-cultures (**c**,**d**), regardless of ascorbic acid (AA) supplementation (*n* = 3 per condition).

In contrast, human SC co-culture with human iPSC-derived MNs, which expressed classic MN markers homeobox 9 (HB9), Islet 1/2, and choline acetyltransferase (ChAT, Supplementary Figure S1), did not exhibit MPZ staining in either the presence or absence of ascorbic acid at either two- or four-week time points (Figure 2).

### 2.2. Human SCs Were Unable to Form Myelinate in Cross-Species Co-Cultures

**Figure 3:**
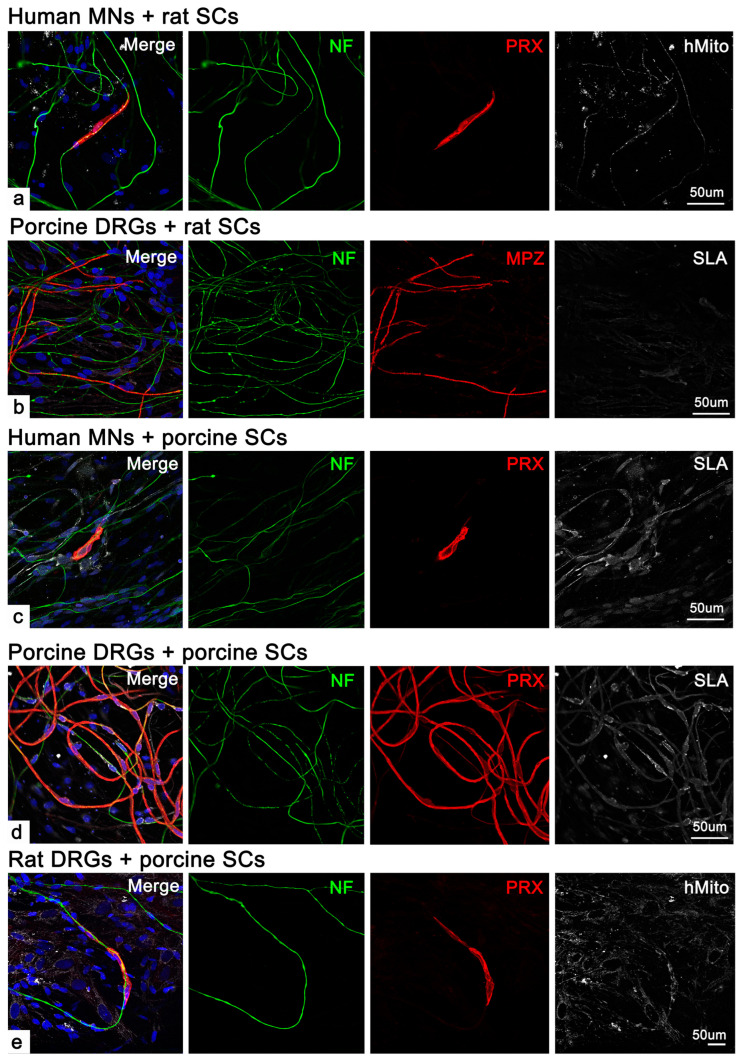
Cross-species myelination by rat or porcine Schwann cells in 2-week co-cultures supplemented with ascorbic acid. Representative image of a rat Schwann cell myelinating a human iPSC-derived motor neuron (MN) neurite identified by a neurofilament (NF) co-labeled with a human mitochondrial marker; rat myelin was labeled with periaxin (PRX) (**a**). Rat Schwann cells were also able to myelinate neurites from porcine dorsal root ganglia (DRG) identified by myelin protein zero (MPZ) staining (**b**). Porcine Schwann cells myelinated neurites from human MNs (**c**) and porcine (**d**) and rat (**e**) DRGs. Porcine myelin was identified by PRX and porcine cells were identified by either swine leukocyte antigen (SLA) when co-cultured with human MNs or porcine DRGs, or human mitochondrial marker antibodies when co-cultured with rat DRGs (**c**–**e**). Quantitative data is included in Table 1.

To determine whether human motor neurons (MNs) differentiated from induced pluripotent stem cells (iPSCs) were receptive to myelination, we performed cross-species co-cultures using human MNs, rat DRGs, or porcine DRGs co-cultured with either human SCs, rat SCs, or porcine SCs (Figure 3). We primarily used PRX to label myelinating SCs due to its positive reactivity in these three species (Figure 3a,c–e), since the MPZ antibody we used was only positive for rat and human cells; however, it was useful for porcine DRGs with rat SC co-cultures (Figure 3b) to rule out any potential porcine SC myelination by residual porcine glial cells. To further distinguish Schwann cells or neurons from different species, we used human mitochondrial markers to identify human and porcine cells, whereas porcine cells were specifically recognized with swine leukocyte antigen (SLA) when co-cultured with human cells (Figure 3). The results showed that both rat and porcine SCs were capable of forming myelin when co-cultured with all three species including human MNs, albeit with much lower occurrence when rat SCs were co-cultured with human MNs or when porcine SCs were co-cultured with rat DRGs. Human SCs failed to form myelin under all conditions (Figure 2 and Figure S2 and Table 1).

### 2.3. Human SCs Largely Failed to Myelinate Mouse Regenerating Axons in Human Nerve Grafts

**Figure 4:**
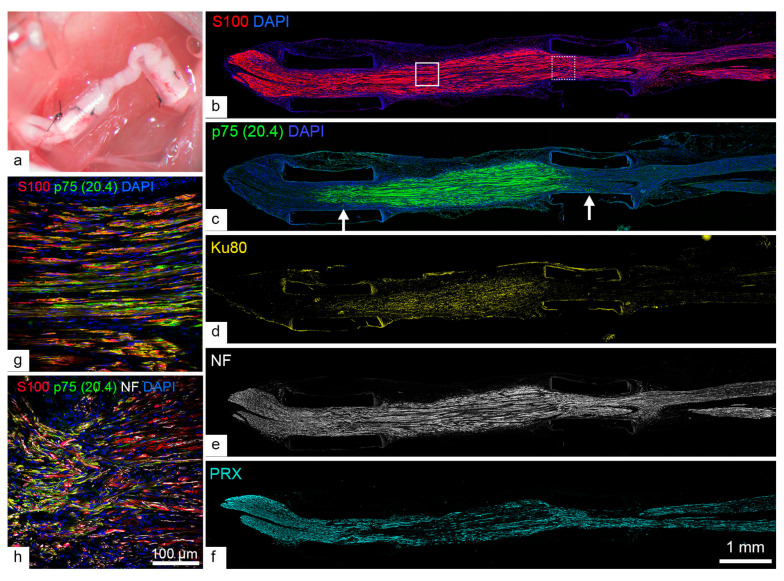
Human sciatic nerve fascicle transplantation in NOD-SCID mice. A 10 mm predegenerated human sciatic nerve fascicle segment was grafted into the NOD-SCID mouse sciatic nerve and splinted with silicone tubes at the coaptation sites (**a**). Immunostaining for pan-SC markers S100 (**b**), p75 (**c**), and Ku80 (**d**) confirms the survival of human Schwann cells. Host axons successfully regenerate into the graft and the distal nerve (**e**), but only limited myelin staining is observed within the human nerve graft segment (**f**). Higher magnification of the central portion ((**g**), solid-line box in (**b**)) and distal end ((**h**), dashed-line box in (**b**)) shows the distribution of primate-specific p75-positive human SCs within the graft and at the coaptation site. The proximal end is shown on the left, with coaptation sites marked by arrows in (**c**).

It is possible that prolonged culture conditions and handling may have adversely affected human SCs, preventing them from forming myelin. We therefore implanted freshly harvested and predegenerated human nerve segments from autopsied donors into NOD-SCID mice, an immunodeficient mouse strain that lacks functional T and B cells in addition to having defective natural killer cell function (Figure 4a). Freshly harvested nerve segments were not exposed to animal serum or proteinase, whereas predegenerated segments were maintained as explants in DMEM with 10% fetal bovine serum and growth factors for two weeks to enhance the number of repair SCs during in vitro degeneration.

Significant human cell loss was found in two of seven transplanted nerves, both from the acutely transplanted group (Supplementary Figure S3). The majority (3/3 predegenerated and 2/4 fresh) of nerve grafts showed good survival of human SCs as indicated by pan-SC marker S100, primate- and porcine-specific p75 (clone ME20.4), and human nuclear marker Ku80 staining (Figure 4b–d and Supplementary Figure S4). Axons regenerated extensively across the grafts into the distal host nerve (Figure 4e and Supplementary Figure S4), though PRX myelin profiles were reduced within the human nerve segment (Figure 4f). We observed no differences in myelination profile or axon regeneration between freshly harvested and predegenerated nerve grafts.

**Figure 5:**
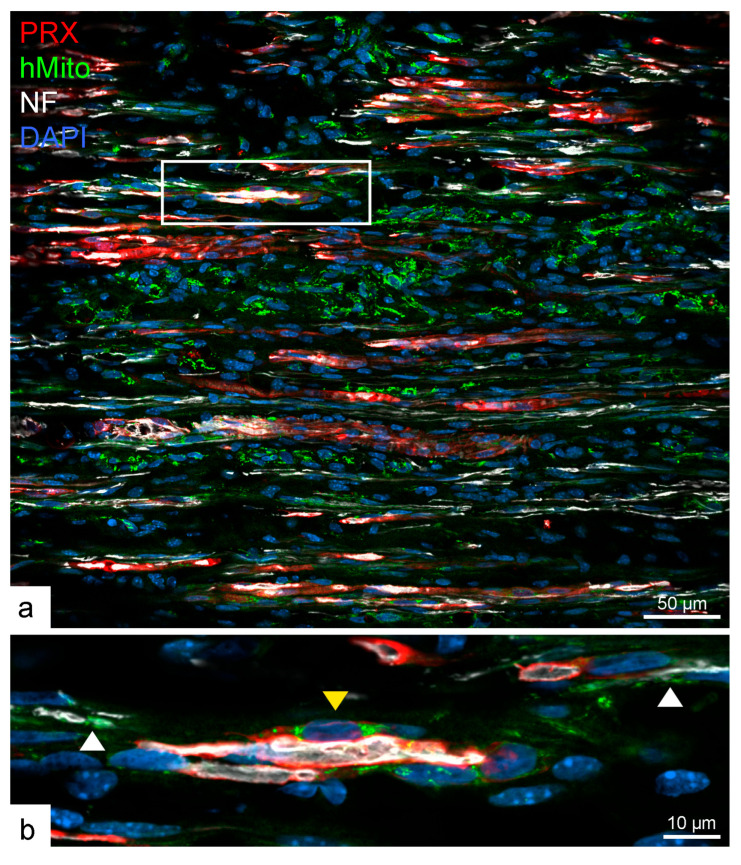
Low- (**a**) and high-magnification (**b**) immunostaining for the myelin marker periaxin (PRX) and the human mitochondrial marker (hMito) reveals a rare instance of a human SC myelinating a mouse axon (yellow arrowhead). A non-myelinating human SC expressing hMito is also seen surrounding an axon (white arrowhead), which is more commonly observed (**b**).

Higher-magnification analysis revealed rare examples of myelinating SCs positive for human mitochondrial markers in their cytoplasm, confirming their human origin. However, the vast majority of myelinating cells were negative for the human marker, suggesting that endogenous host SCs contributed to almost all of the observed myelination (Figure 5).

## 3. Discussion

The present study underscores critical functional differences between human, rodent, and porcine SCs in their capacity to myelinate neurites, both in vitro and in vivo. While rodent and porcine SCs reliably formed myelin in co-culture with sensory neurons or MNs under ascorbic acid-supplemented serum-free media, human SCs consistently failed to myelinate, even when co-cultured with human iPSC-derived MNs or transplanted into regenerating nerves in immunocompromised mice. These findings align with emerging evidence highlighting intrinsic species-specific differences in SC biology [5,8] and emphasize the challenges of translating rodent-derived findings to humans.

SCs have traditionally been harvested from nerves since the development of isolation and purification in the early 1980s [20]. To circumvent the need to sacrifice a healthy donor nerve for clinical use, alternative sources have been sought, many of which have a stem or progenitor cell origin. Specifically, in the human cell model, SCs or SC-like cells have been differentiated from diverse types of tissues including, but not limited to, fibroblasts [21], epidermal neural crest stem cells [17], muscle-derived stem/progenitor cells [22], skin-derived precursors [23,24], dental pulp stem cells [25], umbilical cord- or bone marrow-derived mesenchymal stromal cells [26,27], adipose-derived stem cells [28], pluripotent stem cells [29], and induced pluripotent stem cells [30]. Recently, we have successfully isolated endogenous SCs from skin following the same protocol for nerve-derived SCs with slight modifications [6], mitigating the need to differentiate SCs from multipotent/pluripotent cells, representing another less invasive and safer alternative to nerve-derived SCs. To verify SC functions of these alternative source-derived SCs, in vitro myelination studies have become a crucial component to justify the proper phenotype of the derived cells. Based on our current findings that human SCs inherently show difficulties in myelin formation, it is important to interpret the identity and maturation state of the SCs under study using gene expression results, such as *mpb* and *mpz* expression, as well as histological results, with caution.

Our results support prior studies that demonstrated that human SCs exhibit distinct functional properties compared to rodent SCs [5,8]. In addition, porcine SCs, being phylogenetically closer to humans’, share certain phenotypic characteristics with both species. For example, similar to human SCs, porcine SCs require laminin for proper cell attachment and neuregulin as a trophic factor, though this requirement may be bypassed during co-culture with neurons (unpublished observations). Notably, porcine SCs demonstrated robust myelination when co-cultured with DRGs from their own species (Figure 3d) and exhibited some degree of myelination with both human MNs and rodent DRGs, similar to that with rat SCs (Table 1), which were demonstrated to myelinate human iPSC-derived sensory neurons in fetal bovine serum-supplemented media [11]. However, the myelin segments formed by porcine SCs in human MN co-culture were noticeably shorter than those when porcine SCs were co-cultured with porcine DRGs (Figure 3c). These results indicated that cross-species incompatibility may also be a contributing factor to the deficiency in myelination. It is worth mentioning that these porcine SCs were derived from pig back skin, suggesting that skin-derived SCs also possess the capability to myelinate neurites. To the best of our knowledge, this is the first study to utilize adult porcine DRGs for cell culture and demonstrate extensive neurite outgrowth (Supplementary Figure S5). Given that porcine axons are larger and more comparable in caliber to human axons, the porcine DRG model may serve as a more suitable alternative to rodent models.

The lack of myelination in human SC co-cultures could stem from inadequate axonal signaling or culture conditions optimized for rodent studies. Despite exposing human SC co-cultures to complete myelination medium with addition of 5% fetal bovine serum for one month, no myelin could be found. In addition, supplementation of recombinant neuregulin, an important survival factor and mitogen for Schwann cells [8,31], failed to induce myelination even at a low dose (0.625 nM) that has been shown to promote myelination in rat SCs [32]. The absence of neuregulin is reflected in the tortuous nature of the neurites (Figure 2), which could have resulted from a lack of axonal growth support from healthy SCs. However, inclusion of a standard dose (6.25 nM) of neuregulin also did not induce myelination, as shown in our previous studies [6]. While ascorbic acid alone in serum-free medium is sufficient to induce myelination for rodent and porcine SCs (Figure 1 and Figure 3), human SCs may require additional or distinct cues, such as specific extracellular matrix components (ECM), neuronal activity, or co-stimulation with human-specific growth factors. This is supported by examples of promyelination states of nerve- and skin-derived SCs, characterized by multiple uncompacted membrane layers when these cells were cultured in glycosaminoglycan-enriched collagen nerve conduits, where interaction with the ECM is three-dimensional [6]. Whether human SCs require specific ECM components—for example, laminin 211 [33] or heparan sulfate proteoglycan glypican-1 [34]—or a cocktail of ECM signals for efficient myelination in vitro warrants further investigation. The need for human neuron-specific signals is also exemplified by the overall failure of SCs in fresh and predegenerated human nerve grafts to form myelination in vivo (Figure 4). This is consistent with our previous study that demonstrated purified and cell culture-expanded human SC transplantation in NOD-SCID mice only yielded a limited number of human SCs expressing the myelin marker periaxin [6]. These observations suggest that the lack of myelination is not due to an intrinsic limitation of human SCs after culture but rather an unmet requirement for human-specific signals. Importantly, priming SCs within a predegenerated human nerve segment alone does not appear to be enough to overcome this restriction. Instead, we speculate that human SCs may depend on interactions with human axons or unidentified signals that are absent in murine hosts.

This study has several limitations. Firstly, iPSC-derived MNs may not fully replicate the maturity and complexity of primary human neurons. Particularly, the sizes of the iPSC-derived MN soma and axons are comparatively smaller than those of humans, although cross-sectional electron microscopy images from our previous study showed that many neurites reached the minimum caliber of 1 μm diameter required for SC myelination initiation [6]. It is also worth noting that different differentiation protocols for iPSC-derived MNs yield distinct electrophysiological and phenotypic properties [35], with the current protocol derived from McEachin et al. remaining uncharacterized [36]. However, our co-cultures with in-house differentiation of either iPSC (SCVI-15 line from Stanford Cardiovascular Institute) MNs or previously commercially available MNs (iCell Motor Neurons, [6]) produced the same negative result in SC myelination, suggesting that it is an intrinsic property of human MNs in cell culture conditions. Although our iPSC-derived MN expressed the classic MN markers Islet 1/2, HB9, and ChAT, unfortunately, we did not assess neuregulin expression, which is important for SC myelination [37]. Secondly, the 4-week in vitro and in vivo observation period may have been insufficient for human SC myelination due to the immaturity of iPSC-derived neurons [35] and the time required for human SCs to initiate myelination. While Clark et al. demonstrated myelination of human iPSC-derived sensory neurons at as early as two weeks in vitro [11], other neuronal subtypes may mature differently. For example, human PSC-derived cortical neurons exhibit functional excitatory synapses and spontaneous calcium spikes by 30 days after neurogenesis, progressing gradually to synchronous firing after 40 days [38]. Similarly, Takazawa et al. reported progressive maturation of human embryonic stem cell-derived MNs with more complex neurite outgrowth and repetitive action potential firing by 2 weeks after differentiation; however, the authors noted that their electrophysiological properties remain only comparable to those of newborn rodent MNs [39]. Besides neuronal maturity, human SCs may require prolonged periods to initiate myelination [14], as evidenced by the sparse myelin segments observed in our graft. In contrast to rodent studies, data on human remyelination timelines after nerve injury is limited, and practical challenges precluded definitive ultrastructural analysis. Therefore, it is still unclear when human SCs initiate remyelination of regenerating axons. Taken together, these findings suggest that future studies should incorporate longer observation periods. Thirdly, the delay in tissue processing after postmortem harvesting (<36 h) combined with the two-week explant culture period may expose SCs to prolonged hypoxia and suboptimal culture conditions, potentially altering their functionality. To address this issue, a modified isolation protocol has been developed, which produces highly purified and viable SCs [40]. This is particularly crucial for human SCs, which are prone to senescence in early cultures [5]. While the consensus recommends nerve processing 24 h postmortem to minimize variability [16,41], some studies suggest that extended storage at 4 °C (e.g., several days) does not significantly compromise SC viability and function [41]. However, whether subtle changes occur during delayed tissue processing remains to be determined. Lastly, we did not perform electron microscopy in our human nerve segment transplantation study to confirm mature myelin due to the scarcity of myelinating human SCs. Transmission electron microscopy is still the gold standard to examine myelin formation. A myelinated fiber can be identified by (1) the presence of basal lamina; (2) electron-dense major dense lines with a periodicity of 12–14 nm spacing; and (3) wrapping around an axon greater than 1 µm in diameter, which can be recognized by the presence of neurofilaments, microtubules, and often mitochondria [42] (Supplementary Figure S6a–d). These features are critical for distinguishing myelinated fibers from multilamellar bodies, which are often mistaken for them (Supplementary Figure S6e–g). Future work should optimize culture conditions using human-specific components such as recombinant proteins and growth factors in three-dimensional culture and explore extended timelines. Additionally, single-cell RNA sequencing comparison of human and porcine SCs may offer insights into transcriptional programs that might be needed to initiate myelination.

To conclude, the inability to recapitulate human SC myelination in vitro poses a significant barrier for drug discovery for peripheral nerve repair, where myelination often fails to fully recover. Current models, which rely primarily on rodent SCs, may not predict human responses, underscoring the need for human-specific systems. The growing diversity of human SC sources offers opportunities to explore whether human SCs retain myelination competence under tailored conditions.

## 4. Materials and Methods

### 4.1. Ethical Statement

All animal experiments were approved by the University of Calgary Animal Care Committee (AC20-0172 and AC20-0114). All applicable international, national, and institutional guidelines for the care and use of animals were followed.

### 4.2. Human SC and Motor Neuron Culture

Human SCs were harvested as previously described [6]. Briefly, autopsied nerve samples were acquired via the Southern Alberta Organ Donation Program with donor or family consent. Samples were processed within 36 h (h) from the donors’ time of death. After receipt of tissue samples, they were washed with cold Hanks’ balanced salt solution (HBSS, Gibco, Rockville, MD, USA) three times. Fascicles were then teased from the nerve, cut into 2 mm long pieces, and incubated in complete medium consisting of DMEM with 10% fetal bovine serum (FBS, Canada origin, Gibco, Burlington, ON, Canada), 1% penicillin–streptomycin (P/S, Gibco, USA) supplemented with 50 ng/mL recombinant human NRG1 (100-03, Peprotech, Rocky Hill, NJ, USA), 5 µM forskolin (Fsk, StemCell Tech, Vancouver, BC, Canada), and plasmocin (InvivoGen, San Diego, CA, USA) at 5 µg/mL. Media were changed twice weekly.

After two weeks of explant incubation, the specimens were dissociated in media containing DMEM with 10% FBS, 1.25U dispase (StemCell Tech, Vancouver, BC, Canada), 1.25 mg/mL collagenase IV (Worthington Biochemical Corporation, Lakewood, NJ, USA), and 1% P/S overnight at 37 °C. After passing through a 40 µm mesh (Fisher Scientific, Waltham, MA, USA), cell suspensions were then centrifuged at 1200 rpm (314 g) for 6 min and cells were seeded in poly-D-lysine (PDL, 20 µg/mL in water, Sigma, St. Louis, MO, USA)/laminin (4 µg/mL in DMEM, Sigma, St. Louis, MO, USA)-coated 100 mm dishes with complete medium as described above, except no plasmocin was added. Media were changed twice weekly.

After a week, N-SCs were selected using magnetic cell separation. Briefly, p75 antibody (clone ME20.4, Biolegend, San Diego, CA, USA) and cells were mixed with the EasySep^TM^ Human “Do-it-Yourself” positive selection kit II (StemCell Tech, Vancouver, BC, Canada), and cells expressing p75 receptor were captured and further expanded in PDL- and laminin-coated 100 mm dishes. Cells were used at passages 2–4.

Human motor neurons (MNs) were previously differentiated from induced pluripotent stem cells (iPSCs) with the percentage of MNs at around 25%, as previously described [6,36], and stored in liquid nitrogen. MNs were thawed and co-cultured with SCs in a 1:10 ratio in initial co-culture medium consisting of BrainPhys neuronal medium (StemCell Tech, Vancouver, BC, Canada) with 5% FBS, 1% non-essential amino acid (Gibco, Rockville, MD, USA), 2% SM1 (StemCell Tech, Vancouver, BC, Canada), 1% N2A (StemCell Tech, Vancouver, BC Canada), 0.2% P/S, 1% Glutamax, and 110 µM 2-mercapoethanol (Gibco, Rockville, MD, USA). Cells were seeded in 12 mm PDL- and laminin-coated coverslips in 24-well plates or 96-well plates.

### 4.3. Rodent SC and Dorsal Root Ganglion (DRG) Cell Culture

Rat sciatic nerve SCs were harvested from 3-month-old Sprague Dawley rats (*n* = 5, Charles River, St. Constant, QC, Canada). After animals were euthanized with sodium pentobarbital (Euthanyl), their sciatic nerves were exposed in both thighs and a 2 cm long nerve from each side was harvested and collected in DMEM. Epineurium was removed and the nerve was washed in DMEM three times before being cut into 2 mm length pieces and incubated as a tissue explant in complete medium as described above. The procedure and medium used in isolating the SCs were identical to those for human SCs, except antibody against rat p75 (Clone MC192, Biosensis, Thebarton, SA, Australia) was used in magnetic cell separation.

Rat DRG cells were harvested from 3-month-old Sprague Dawley rats (*n* = 4). After animals were euthanized, cervical and lumbar enlargement DRGs were collected as described previously [43]. Briefly, approximately 16 DRGs were harvested from each rat and incubated at 0.0625% collagenase for 2 h at 37 °C followed by washing twice with co-culture medium. The cells were then layered on a 15% bovine serum album cushion and centrifuged at 200× *g* for 10 min to remove non-neuronal cells and debris. The pellet was resuspended in warm initial co-culture medium, counted, and mixed with rat SCs at a ratio of 1:10 before seeding in 12 mm PDL- and laminin-coated coverslips in 24-well plates or 96-well plates.

### 4.4. Porcine SC and Dorsal Root Ganglion Cell Culture

Porcine cells were harvested from secondary use of tissues from a separate protocol. No pigs were killed solely for this study.

Porcine skin-derived SCs were acquired using the same method as the human skin-derived SC harvesting protocol as published previously [6]. Briefly, 2 cm^2^-sized back skin from 7-month-old KuneKune minipigs (local farm, Alberta, Canada) was harvested, the epidermis removed and cut into small pieces (2 × 2 mm), then incubated as an explant culture similar to human nerve-derived SCs as described above. Cell medium, tissue dissociation, purification, and passages were identical to those for human nerve-derived SCs.

Porcine cervical dorsal root ganglion cells were harvested from 9-month-old KuneKune minipigs (which had undergone peroneal nerve injuries in a separate study) and the procedures were identical to those of rat DRGs, except the DRGs were incubated in 0.0625% collagenase IV for 3 h at 37 °C.

### 4.5. Co-Culture Treatments

After 2 days in initial co-culture medium, the medium was changed to serum-free myelination co-culture medium consisting of BrainPhys neuronal medium, 1% non-essential amino acid, 2% SM1 (StemCell Tech, Vancouver, BC, Canada), 1% N2A (StemCell Tech, Vancouver, BC, Canada), 0.2% P/S, ascorbic acid (50 µg/mL, Sigma, USA), and growth factor-reduced Matrigel (1:200, Corning, New York, NY, USA). Media were changed every two to three days. Co-cultures were kept for two to four weeks after myelination induction.

### 4.6. Sciatic Nerve Injury, Human Nerve Transplantation, Tissue Harvest, and Immunohistochemistry

NOD-SCID mice (NOD.CB17*-Prkdc^scid^*/NCrCrl, code: 394, Charles River, St. Constant, QC, Canada) at 4–5 months were used to receive human nerve fascicle transplantation. Upon receipt of human autopsied sciatic nerve samples, nerves were washed extensively in DMEM and cut into 1.5 cm segments. Fascicles comparable in size to mouse sciatic nerves were then pulled from the nerve segment using a pair of #5 forceps and stored on ice in DMEM until the animals were ready. Four mice received acutely transplanted human nerves, and three mice received predegenerated nerves. For nerve predegeneration, the 1.5 cm long fascicles were incubated as explants in DMEM with 10% FBS, 1% P/S, supplemented with 50 ng/mL NRG1, 5 µM forskolin, and 5 µg/mL plasmocin for two weeks. Media were changed twice weekly before animal surgery. Briefly, animals were deeply anesthetized using 1.5% isoflurane with oxygen, and the surgical area was then shaved and disinfected with betadine and isopropanol. Buprenorphine (0.05 mg/ kg, s.c.) and meloxicam (1 mg/kg, s.c.) were given before surgery. Under the aseptic technique, the sciatic nerve was then exposed, and a 5 mm segment of the sciatic nerve was then removed. The human nerve fascicle cut to a 10 mm length was transplanted into the nerve gap and secured in place with multiple 10-0 sutures and splinted with a sterile silicone tube (internal diameter: 0.64 mm) at each neurorrhaphy site. The incision was closed with 6-0 prolene sutures and the animal was returned to its home cage for recovery on a heating pad. Post-operative care included meloxicam injection (1 mg/kg, s.c. daily) for two days after surgery.

Animals were euthanized after surviving for four weeks using isoflurane followed by overdosed sodium pentobarbital (Euthanyl). Transplanted human nerve fascicles with host sciatic nerve ends were harvested and immerse-fixed in 4% paraformaldehyde (PFA) overnight at 4 °C and subsequently placed in 30% phosphate-buffered sucrose until sectioning. After the samples had sunk, silicone splints were removed and nerve samples were embedded in optimum cutting temperature compound (VWR, Radnor, PA, USA), frozen in pre-cooled isopentane, and cut into longitudinal sections at a thickness of 10 μm on a cryostat. The sections were collected on SuperFrosted Plus slides (VWR, Radnor, PA, USA) and stored at −20 °C for later use.

### 4.7. Nile Red Labeling, Immunocytochemistry, and Immunohistochemistry

For Nile red myelin labeling, cells were fixed with 4% PFA for 10 min at room temperature, washed three times with 0.01 M phosphate-buffered saline (PBS), and incubated with 10 μM Nile red (Sigma, USA) for 10 min at room temperature. Cells were washed three times with 0.01 M PBS and mounted in PermaFluor mounting medium (Thermo Scientific, Waltham, MA, USA). For immunocytochemistry, cells were fixed with 4% PFA for 10 min at room temperature, washed three times with 0.01 M PBS, and blocked with 5% normal donkey serum (NDS) for 1 h at room temperature. Primary antibodies (Table 2) included rabbit anti-periaxin (PRX, 1:500, Novus Biologicals, # NBP-1-89598, Littleton, CO, USA), rabbit anti-myelin protein zero (MPZ, 1:500, Abcam, # ab31851, Cambridge, UK), chicken anti-neurofilament 200 (1:10,000, BioLegend, # 822601, San Diego, CA, USA), mouse anti-human mitochondria (1:500, Millipore, #MAB1273, Billerica, MA, USA), mouse anti-swine leukocyte antigen-Class II (1:200, Bio-Rad, MCA2314GA, Hercules, CA, USA), mouse anti-HB9 (1:200, Developmental Studies Hybridoma Bank, 81.5C10, Iowa City, IA, USA), mouse anti-Islet 1/2 (1:200, Developmental Studies Hybridoma Bank, 39.4D5, Iowa City, IA, USA), and goat anti-choline acetyltransferase (ChAT, 1:500, Sigma, AB144P, St. Louis, MO, USA). Primary antibodies were omitted in secondary-only controls. After three washes (15 min each) in PBS–Tween 20 (0.05%, PBS-T), the cells were incubated in the corresponding fluorescent conjugated secondary antibodies (1:500, Invitrogen, Carlsbad, CA, USA) in PBS for 1 h at RT. After 3 washes in PBS-T, coverslips were mounted with PermaFluor mounting medium and sealed with nail polish.

For immunohistochemistry, sections were washed with PBS and blocked with 5% NDS, followed by incubation with additional primary antibodies which included mouse anti-primate and porcine-specific p75 (in-house-made hybridoma supernatant from the 200-3-G6-4 cell line), rabbit anti-S100 (1:6, Dako, # Z0311, Glostrup, Denmark), and rabbit anti-Ku80 (1:200, Cell Signaling, #2180, Danvers, MA, USA). Sections were then washed with PBST three times followed by corresponding secondary antibodies and mounted with mounting medium.

### 4.8. Imaging, Myelin Segment Quantification, and Statistical Analysis

For coverslips and tissue slides, images were captured using a Nikon A1R confocal microscope (Nikon, Tokyo, Japan) equipped with a 25× lens (NA = 1.1). For 96-well plates, images were captured using an ImageXpress Micro XLS with a 10× lens (Molecular Devices, San Jose, CA, USA) or a Lecia SP8 confocal microscope with a 25× lens (Leica, Wetzlar, Germany). All myelin segments were manually quantified from images acquired using ImageXpress, with counts performed in three 12 mm coverslips per condition in 24-well plates or across 7 wells per condition in 96-well plates. This analysis was conducted in triplicate as independent experiments; the average number is expressed as mean ± SEM. Data was analyzed with Prism 10 software (GraphPad, San Diego, CA, USA) using Student’s *t*-test or one-way ANOVA to compare between groups. Statistical significance was accepted at *p* < 0.05.

## Figures and Tables

**Table 1:** Average number of myelin segments per well (*n* = 7) in 96-well plate when rat, human, or porcine neurons were co-cultured with rat, human, or porcine Schwann cells. Statistical comparisons were performed using one-way ANOVA with Dunnett’s post hoc test, with all conditions compared against human Schwann cell co-cultures for each type of neurons. * *p* < 0.05; ** *p* < 0.01; ***, *p* < 0.001.

Co-Culture With	Human SCs	Rat SCs	Porcine SCs
Rat DRGs	Nil	13.3 ± 3.5 ***	0.6 ± 0.4
Human MNs	Nil	2 ± 0.9	4.4 ± 1.6 *
Porcine DRGs	Nil	70 ± 13.2 **	67.1 ± 20.6 **

**Table 2:** Primary antibodies used, their dilutions, and their purposes for this study.

Primary Antibody	Brand	Catalog Number	Dilution	Purpose
Rabbit anti-periaxin (PRX)	Novus Biologicals	NBP-1-89598	1:500	To label myelinating Schwann cells (all species *)
Rabbit anti-myelin protein zero (MPZ)	Abcam	ab31851	1:500	To identify rodent and human myelin segments, negative in porcine myelin
Chicken anti-neurofilament 200	BioLegend	822601	1:10,000	To label neuronal processes (all species *)
Mouse anti-human mitochondria	Millipore	MAB1273	1:500	To identify human and porcine cells
Mouse anti-swine leukocyte antigen-Class II	Bio-Rad	MCA2314GA	1:200	To label porcine cells
Mouse anti-primate and porcine-specific p75	N/A	Hybridoma supernatant	As is	To identify human and porcine Schwann cells
Rabbit anti-S100	Dako	Z0311	1:6	To label Schwann cells (all species *)
Rabbit anti-Ku80	Cell Signaling	2180	1:200	To identify human nuclei
Mouse anti-HB9	DSHB	81.5C10	1:200	To identify motor neurons
Mouse anti-Islet 1/2	DSHB	39.4D5	1:200	To identify motor neurons
Goat anti-choline acetyltransferase	Sigma	AB144P	1:500	To identify motor neurons

* All species means rodents, humans, and pigs.

## Data Availability

The original contributions presented in this study are included in the article/Supplementary Materials. Further inquiries can be directed to the corresponding author.

## Supplementary Materials

The following supporting information can be downloaded at: https://www.mdpi.com/article/10.3390/ijms26136457/s1.

## References

[1] Jessen K.R., Mirsky R. (2019). The Success and Failure of the Schwann Cell Response to Nerve Injury. Front. Cell. Neurosci..

[2] Anderson K.D., Guest J.D., Dietrich W.D., Bartlett Bunge M., Curiel R., Dididze M., Green B.A., Khan A., Pearse D.D., Saraf-Lavi E. (2017). Safety of Autologous Human Schwann Cell Transplantation in Subacute Thoracic Spinal Cord Injury. J. Neurotrauma.

[3] Gersey Z.C., Burks S.S., Anderson K.D., Dididze M., Khan A., Dietrich W.D., Levi A.D. (2017). First Human Experience with Autologous Schwann Cells to Supplement Sciatic Nerve Repair: Report of 2 Cases with Long-Term Follow-Up. Neurosurg. Focus.

[4] Bianchini D., De Martini I., Cadoni A., Zicca A., Tabaton M., Schenone A., Anfosso S., Wattar A.S.A., Zaccheo D., Mancardi G.L. (1992). GFAP Expression of Human Schwann Cells in Tissue Culture. Brain Res..

[5] Weiss T., Taschner-Mandl S., Bileck A., Slany A., Kromp F., Rifatbegovic F., Frech C., Windhager R., Kitzinger H., Tzou C.H. (2016). Proteomics and Transcriptomics of Peripheral Nerve Tissue and Cells Unravel New Aspects of the Human Schwann Cell Repair Phenotype. Glia.

[6] Chu T., Baral K., Labit E., Rosin N., Sinha S., Umansky D., Alzahrani S., Arora R., Cao L., Rancourt D. (2022). Comparison of Human Skin- and Nerve-Derived Schwann Cells Reveals Many Similarities and Subtle Genomic and Functional Differences. Glia.

[7] Mathon N.F., Malcolm D.S., Harrisingh M.C., Cheng L., Lloyd A.C. (2001). Lack of Replicative Senescence in Normal Rodent Glia. Science.

[8] Monje P.V., Sant D., Wang G. (2018). Phenotypic and Functional Characteristics of Human Schwann Cells as Revealed by Cell-Based Assays and RNA-SEQ. Mol. Neurobiol..

[9] Monje P.V. (2020). The Properties of Human Schwann Cells: Lessons from In Vitro Culture and Transplantation Studies. Glia.

[10] Chan J.R., Watkins T.A., Cosgaya J.M., Zhang C., Chen L., Reichardt L.F., Shooter E.M., Barres B.A. (2004). NGF Controls Axonal Receptivity to Myelination by Schwann Cells or Oligodendrocytes. Neuron.

[11] Clark A.J., Kaller M.S., Galino J., Willison H.J., Rinaldi S., Bennett D.L.H. (2017). Co-Cultures with Stem Cell-Derived Human Sensory Neurons Reveal Regulators of Peripheral Myelination. Brain.

[12] Levi A.D., Guenard V., Aebischer P., Bunge R.P. (1994). The Functional Characteristics of Schwann Cells Cultured from Human Peripheral Nerve after Transplantation into a Gap within the Rat Sciatic Nerve. J. Neurosci..

[13] Levi A.D.O., Bunge R.P. (1994). Studies of Myelin Formation after Transplantation of Human Schwann Cells into the Severe Combined Immunodeficient Mouse. Exp. Neurol..

[14] Morrissey T.K., Kleitman N., Bunge R.P. (1995). Human Schwann Cells in Vitro. II. Myelination of Sensory Axons Following Extensive Purification and Heregulin-Induced Expansion. J. Neurobiol..

[15] Majd H., Amin S., Ghazizadeh Z., Cesiulis A., Arroyo E., Lankford K., Majd A., Farahvashi S., Chemel A.K., Okoye M. (2023). Deriving Schwann Cells from hPSCs Enables Disease Modeling and Drug Discovery for Diabetic Peripheral Neuropathy. Cell Stem Cell.

[16] Bastidas J., Athauda G., De La Cruz G., Chan W.M., Golshani R., Berrocal Y., Henao M., Lalwani A., Mannoji C., Assi M. (2017). Human Schwann Cells Exhibit Long-Term Cell Survival, Are Not Tumorigenic and Promote Repair When Transplanted into the Contused Spinal Cord. Glia.

[17] Sakaue M., Sieber-Blum M. (2015). Human Epidermal Neural Crest Stem Cells as a Source of Schwann Cells. Development.

[18] Walters E.M., Wells K.D., Bryda E.C., Schommer S., Prather R.S. (2017). Swine Models, Genomic Tools and Services to Enhance Our Understanding of Human Health and Diseases. Lab Anim..

[19] Grochmal J., Teo W., Gambhir H., Kumar R., Stratton J.A., Dhaliwal R., Brideau C., Biernaskie J., Stys P.K., Midha R. (2019). A Novel Approach to 32-Channel Peripheral Nervous System Myelin Imaging In Vivo, with Single Axon Resolution. J. Neurosurg..

[20] Monje P.V. (2020). Schwann Cell Cultures: Biology, Technology and Therapeutics. Cells.

[21] Mazzara P.G., Massimino L., Pellegatta M., Ronchi G., Ricca A., Iannielli A., Giannelli S.G., Cursi M., Cancellieri C., Sessa A. (2017). Two Factor-Based Reprogramming of Rodent and Human Fibroblasts into Schwann Cells. Nat. Commun..

[22] Lavasani M., Thompson S.D., Pollett J.B., Usas A., Lu A., Stolz D.B., Clark K.A., Sun B., Péault B., Huard J. (2014). Human Muscle–Derived Stem/Progenitor Cells Promote Functional Murine Peripheral Nerve Regeneration. J. Clin. Investig..

[23] Krause M.P., Dworski S., Feinberg K., Jones K., Johnston A.P.W., Paul S., Paris M., Peles E., Bagli D., Forrest C.R. (2014). Direct Genesis of Functional Rodent and Human Schwann Cells from Skin Mesenchymal Precursors. Stem Cell Rep..

[24] Biernaskie J.A., McKenzie I.A., Toma J.G., Miller F.D. (2006). Isolation of Skin-Derived Precursors (SKPs) and Differentiation and Enrichment of Their Schwann Cell Progeny. Nat. Protoc..

[25] Martens W., Sanen K., Georgiou M., Struys T., Bronckaers A., Ameloot M., Phillips J., Lambrichts I. (2014). Human Dental Pulp Stem Cells Can Differentiate into Schwann Cells and Promote and Guide Neurite Outgrowth in an Aligned Tissue-Engineered Collagen Construct in Vitro. FASEB J..

[26] Cai S., Tsui Y.-P., Tam K.-W., Shea G.K.-H., Chang R.S.-K., Ao Q., Shum D.K.-Y., Chan Y.-S. (2017). Directed Differentiation of Human Bone Marrow Stromal Cells to Fate-Committed Schwann Cells. Stem Cell Rep..

[27] Matsuse D., Kitada M., Kohama M., Nishikawa K., Makinoshima H., Wakao S., Fujiyoshi Y., Heike T., Nakahata T., Akutsu H. (2010). Human Umbilical Cord-Derived Mesenchymal Stromal Cells Differentiate Into Functional Schwann Cells That Sustain Peripheral Nerve Regeneration. J. Neuropathol. Exp. Neurol..

[28] Tomita K., Madura T., Sakai Y., Yano K., Terenghi G., Hosokawa K. (2013). Glial Differentiation of Human Adipose-Derived Stem Cells: Implications for Cell-Based Transplantation Therapy. Neuroscience.

[29] Kim H.S., Lee J., Lee D.Y., Kim Y.D., Kim J.Y., Lim H.J., Lim S., Cho Y.S. (2017). Schwann Cell Precursors from Human Pluripotent Stem Cells as a Potential Therapeutic Target for Myelin Repair. Stem Cell Rep..

[30] Liu Q., Spusta S.C., Mi R., Lassiter R.N.T., Stark M.R., Höke A., Rao M.S., Zeng X. (2012). Human Neural Crest Stem Cells Derived from Human ESCs and Induced Pluripotent Stem Cells: Induction, Maintenance, and Differentiation into Functional Schwann Cells. Stem Cells Transl. Med..

[31] Birchmeier C., Nave K. (2008). Neuregulin-1, a Key Axonal Signal That Drives Schwann Cell Growth and Differentiation. Glia.

[32] Zanazzi G., Einheber S., Westreich R., Hannocks M.J., Bedell-Hogan D., Marchionni M.A., Salzer J.L. (2001). Glial Growth Factor/Neuregulin Inhibits Schwann Cell Myelination and Induces Demyelination. J. Cell Biol..

[33] McKee K.K., Yang D.-H., Patel R., Chen Z.-L., Strickland S., Takagi J., Sekiguchi K., Yurchenco P.D. (2012). Schwann Cell Myelination Requires Integration of Laminin Activities. J. Cell Sci..

[34] Chernousov M.A., Rothblum K., Stahl R.C., Evans A., Prentiss L., Carey D.J. (2006). Glypican-1 and A4(V) Collagen Are Required for Schwann Cell Myelination. J. Neurosci..

[35] Sances S., Bruijn L.I., Chandran S., Eggan K., Ho R., Klim J.R., Livesey M.R., Lowry E., Macklis J.D., Rushton D. (2016). Modeling ALS with Motor Neurons Derived from Human Induced Pluripotent Stem Cells. Nat. Neurosci..

[36] McEachin Z.T., Gendron T.F., Raj N., García-Murias M., Banerjee A., Purcell R.H., Ward P.J., Todd T.W., Merritt-Garza M.E., Jansen-West K. (2020). Chimeric Peptide Species Contribute to Divergent Dipeptide Repeat Pathology in c9ALS/FTD and SCA36. Neuron.

[37] Stassart R.M., Fledrich R., Velanac V., Brinkmann B.G., Schwab M.H., Meijer D., Sereda M.W., Nave K.A. (2013). A Role for Schwann Cell-Derived Neuregulin-1 in Remyelination. Nat. Neurosci..

[38] Ciceri G., Baggiolini A., Cho H.S., Kshirsagar M., Benito-Kwiecinski S., Walsh R.M., Aromolaran K.A., Gonzalez-Hernandez A.J., Munguba H., Koo S.Y. (2024). An Epigenetic Barrier Sets the Timing of Human Neuronal Maturation. Nature.

[39] Takazawa T., Croft G.F., Amoroso M.W., Studer L., Wichterle H., MacDermott A.B. (2012). Maturation of Spinal Motor Neurons Derived from Human Embryonic Stem Cells. PLoS ONE.

[40] Andersen N.D., Srinivas S., Piñero G., Monje P.V. (2016). A Rapid and Versatile Method for the Isolation, Purification and Cryogenic Storage of Schwann Cells from Adult Rodent Nerves. Sci. Rep..

[41] Aparicio G., Monje P. (2023). Human Schwann Cells in Vitro I. Nerve Tissue Processing, Pre-Degeneration, Isolation, and Culturing of Primary Cells. BIO-Protocol.

[42] Kidd G.J., Ohno N., Trapp B.D. (2013). Biology of Schwann Cells. Handbook of Clinical Neurology.

[43] Owen D.E., Egerton J., Skaper S.D. (2012). Culture of Dissociated Sensory Neurons from Dorsal Root Ganglia of Postnatal and Adult Rats. Neurotrophic Factors: Methods and Protocols.

