# Supplementary material for: Limited Myelination Capacity in Human Schwann Cells in Experimental Models in Comparison to Rodent and Porcine Schwann Cells
Source: Int J Mol Sci. 2025 Jul 4;26(13):6457. doi: 10.3390/ijms26136457 (PMC12250475; doi:10.3390/ijms26136457)
Supplement: Supplementary file 1 [file ijms-26-06457-s001.zip › ijms-3674293-supplementary.pdf]

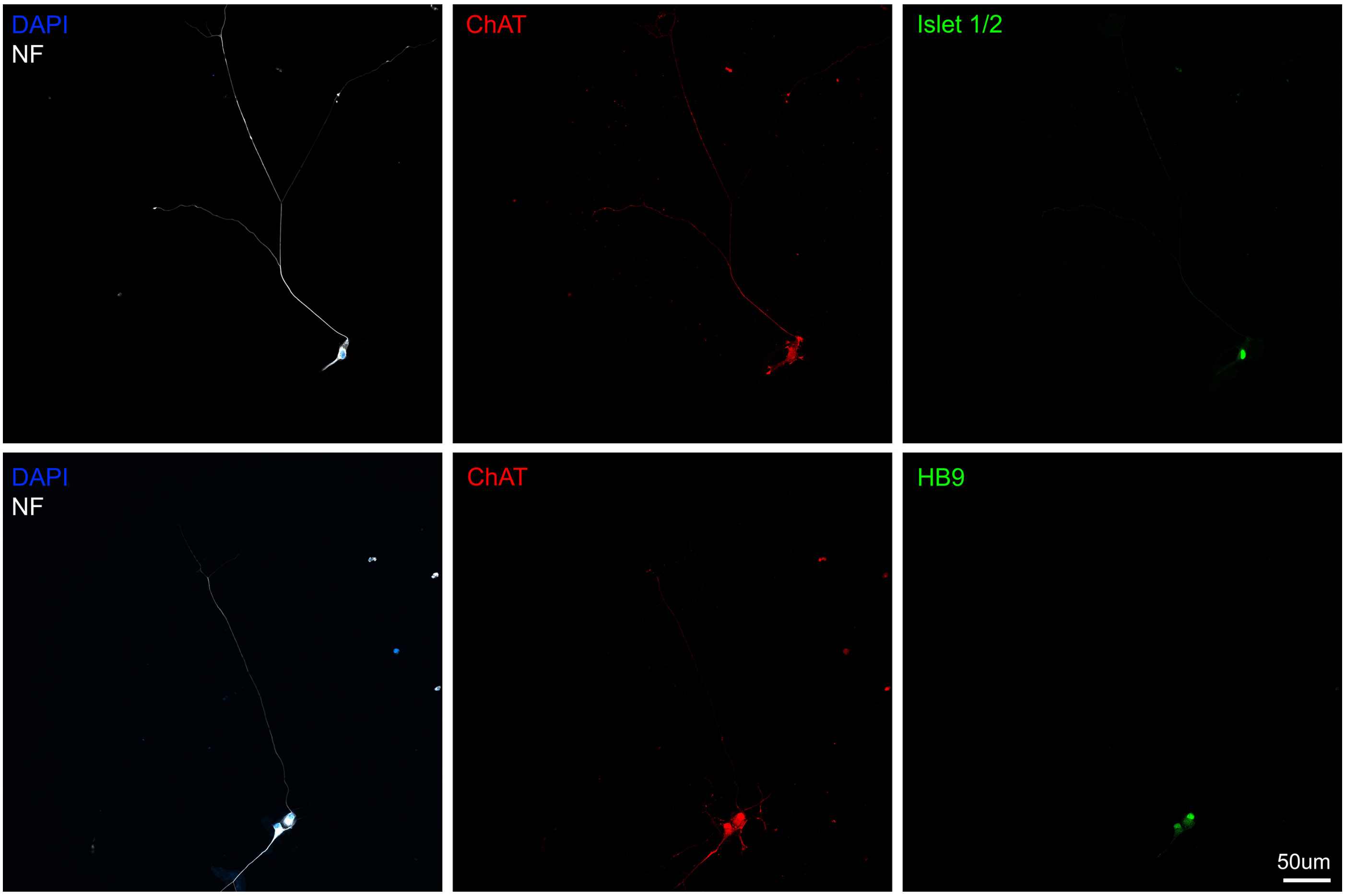

Supp. Figure S1

iPSC-derived motor neurons cultured for seven days on poly-D-lysine- and laminin-coated substrate expressed classic motor neuron markers choline acetyltransferase (ChAT), which colocalized with neurofilament (NF), and HB9 and Islet 1/2, which were in DAPI-labeled nuclei.

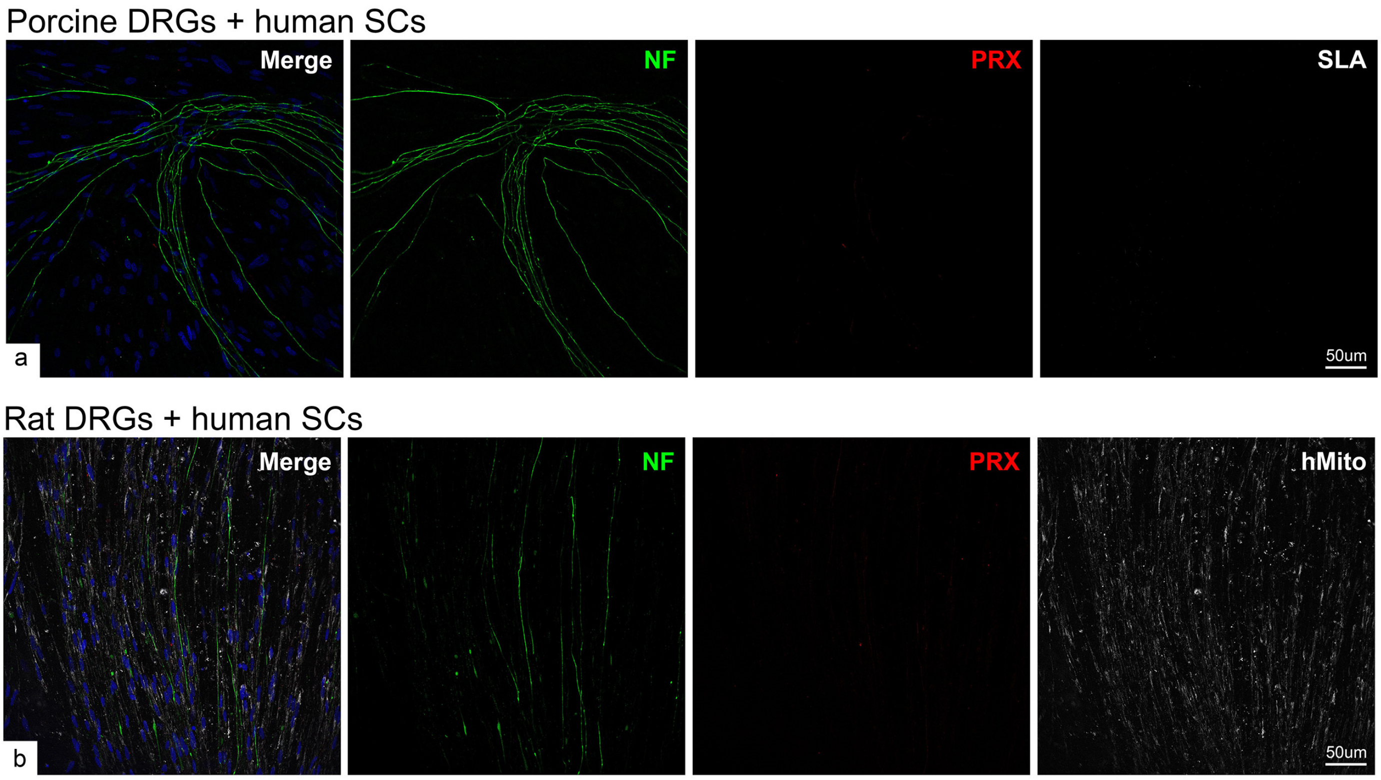

Supp. Figure S2

Absence of human Schwann cell myelination when co-cultured with porcine dorsal root ganglion (DRG, a) or rat DRG cells in 2-week cultures supplemented with ascorbic acid. Neurites were labeled with a neurofilament (NF) and myelin was labeled with periaxin (PRX) antibodies. Unlike porcine Schwann cells which expressed high levels of swine leukocyte antigen (SLA; see Figure 3), porcine neurons did not express detectable SLA. The absence of SLA suggested no contamination from porcine glial cells. Human Schwann cells were identified using a human mitochondrial marker (hMito) in co-cultures with rat DRGs.

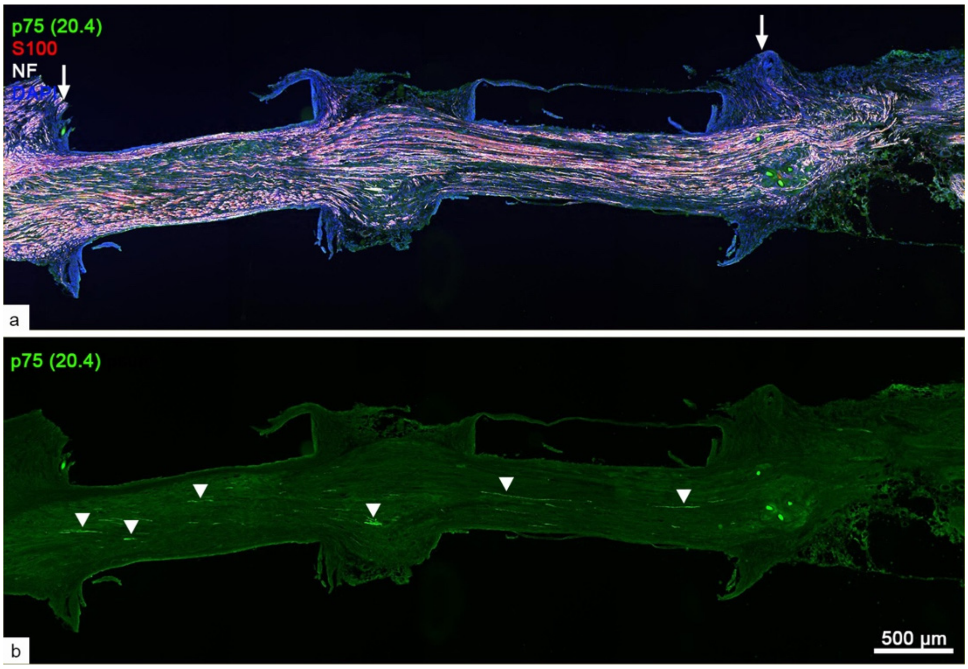

Supp. Figure S3

Limited survival of human Schwann cells in a freshly harvested human sciatic nerve graft. The proximal end is shown on the left, with coaptation sites marked by arrows (a). Numerous S100-positive Schwann cells were present in the human nerve segment (a) but only a few were positive for primate-specific p75 (20.4) antibodies (arrowheads in b).

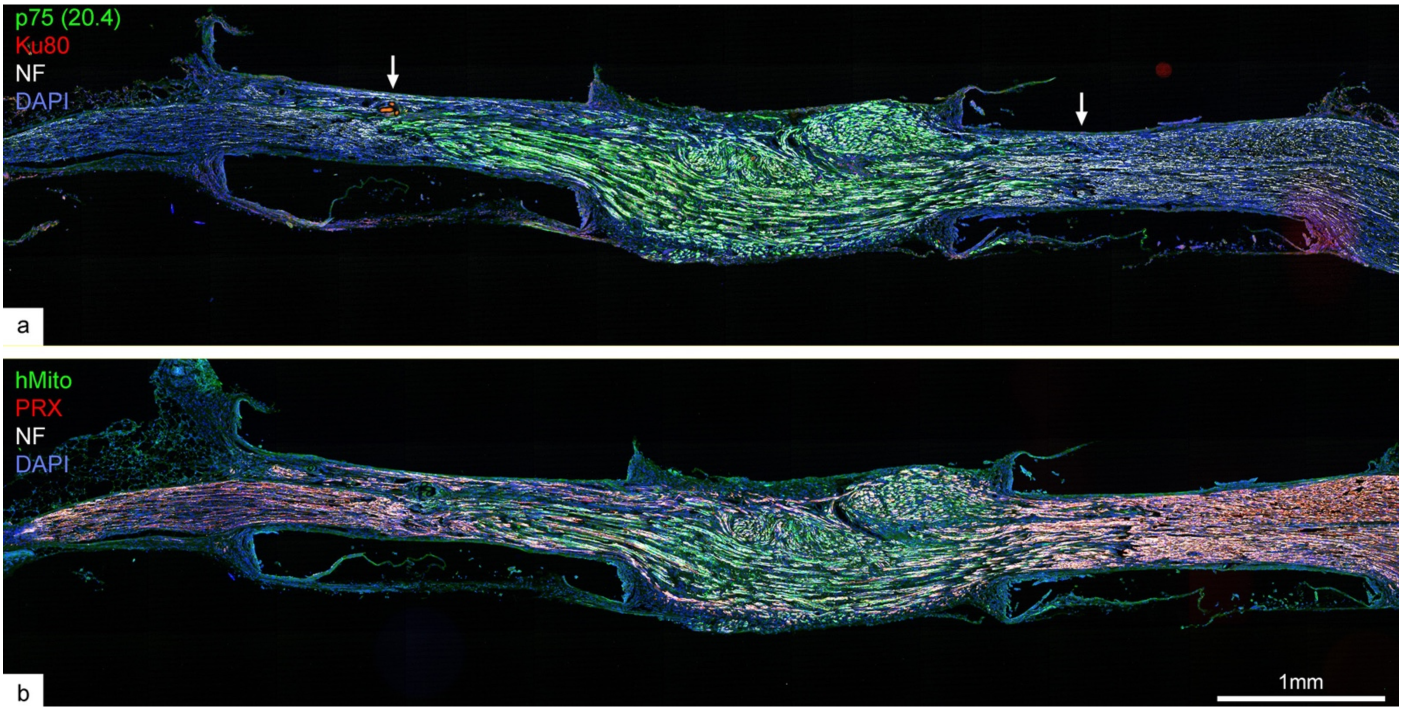

Supp. Figure S4

Retention of human cells in a freshly harvested human sciatic nerve graft, identified using p75 and Ku80 staining (a). Myelination is primarily observed in the host nerve and the outer portion of the human nerve graft, with most periaxin (PRX)-positive myelinating cells being endogenous mouse Schwann cells that do not express the human mitochondrial marker (20.4) (b). The proximal end is shown on the left, with coaptation sites marked by arrows in (a).

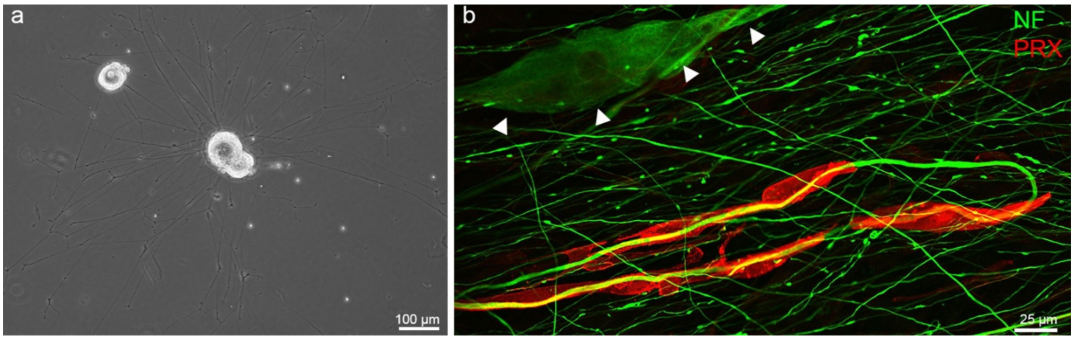

Supp. Figure S5

Porcine dorsal root ganglion cells exhibited extensive neurite outgrowth two days after seeding on a poly-lysine- and laminin-coated substrate, as shown under a phase-contrast microscope (a). A confocal image shows a large-caliber (>2 µm diameter) porcine neurite (neurofilament, NF) being myelinated by porcine Schwann cells (periaxin, PRX-positive) in co-culture. A porcine dorsal root ganglion cell body (arrowheads) is visible adjacent to the myelinated fiber (b).

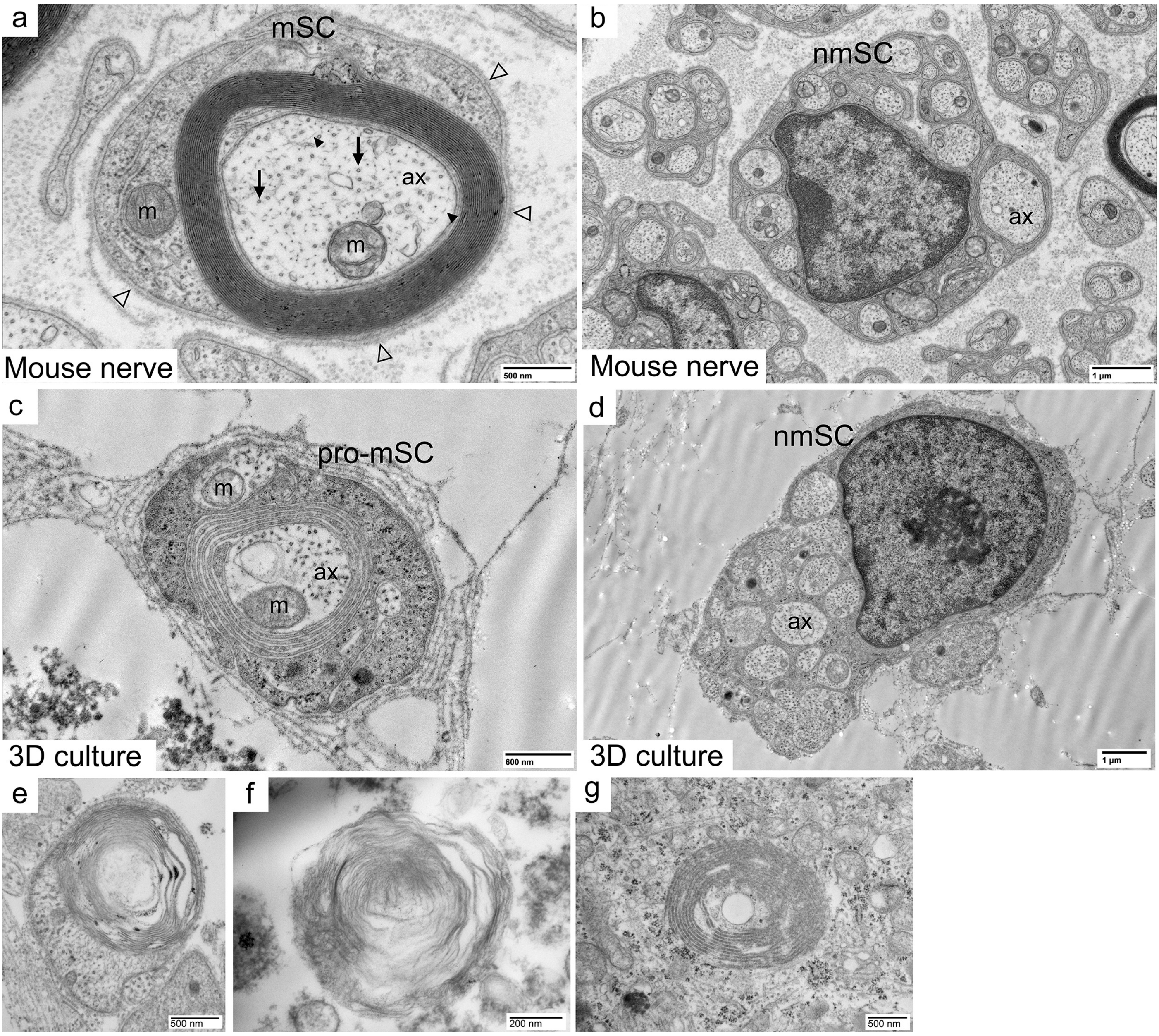

Supp. Figure S6

Transmission electron microscopy of myelinating and non-myelinating Schwann cells in experimental models. Myelinating Schwann cells (mSC, a) and non-myelinating Schwann cells (nmSC, b) are shown in naïve mouse nerves. The myelin sheath exhibits periodicity, wrapping around an axon (ax) containing mitochondria (m), microtubules (arrows), and neurofilaments (arrowheads). A layer of basal lamina (open arrowheads) also surrounds the Schwann cell body. Promyelinating (pro-mSC, c) and non-myelinating (d) human Schwann cells in three-dimensional (3D) co-culture are shown from our previous study, no compact myelin was found. Multilamellar bodies (e-f) are often mistaken for myelin structures. These structures can be found within an axon (e), in the extracellular matrix (f), and inside a fibroblast (g), respectively.
